# Violent Behavior Prior to Admission Is Not a Factor in Further Prolonged Length of Stay: A Retrospective Cohort Study in a Japanese Psychiatric Hospital

**DOI:** 10.3389/fpsyt.2021.600456

**Published:** 2021-07-05

**Authors:** Hidetoshi Kinoshita, Noriomi Kuroki, Takayuki Okada

**Affiliations:** ^1^Department of Psychiatry, Tokyo Metropolitan Matsuzawa Hospital, Tokyo, Japan; ^2^Forensic Mental Health Unit, Department of Psychiatry and Behavioral Sciences, Division of Cognitive and Behavioral Medicine, Graduate School of Medical and Dental Sciences, Tokyo Medical and Dental University, Tokyo, Japan

**Keywords:** length of stay, deinstitutionalization, general psychiatry, Asia, Japan, violent behavior

## Abstract

**Background:** This study assessed the hypothesis that violent behavior prior to admission prolongs psychiatric hospitalization and evaluated the likelihood of hospital discharge to a community care setting based on demographic and clinical factors, with an emphasis on violent behavior.

**Methods:** We retrospectively selected 362 patients who were involuntarily admitted to a psychiatric hospital in Japan from December 1, 2015 to November 30, 2017, stayed longer than planned, and underwent review by a multidisciplinary team. We assessed (a) education and marital status and history of substance abuse, (b) the presence/absence and type of violent behavior that led to hospital admission, and (c) the discharge criteria. We divided the subjects into groups according to whether they had demonstrated violent behavior prior to admission and compared demographic and clinical variables between the groups using bivariate analysis. We also analyzed data using the Cox proportional hazard model, defining discharge to the community as the outcome. Age, sex, and variables that were significant at a level of *P* < 0.05 based on Cox univariate analysis were included in the multivariate models using the forced entry method.

**Results:** The Violent group included 94 patients (26%). There were no significant between-group differences in age, sex, educational background, marital history, career history, or the history of substance abuse. However, hospitalization was significantly longer in the Non-violent group. The Cox proportional multivariate hazard ratios revealed that violent behavior prior to admission resulted in a higher probability of hospital discharge.

**Conclusion:** Violent behavior prior to admission did not significantly contribute to prolonged hospitalization in patients who deviated from the treatment plan and had exceeded the planned hospitalization duration. Our findings recommend caution when using violence and impulsiveness observed during the acute stage to predict the difficulty of long-term treatment.

## Introduction

“Deinstitutionalization,” or promoting patient-centered psychiatric treatment by transferring the site of treatment to the community, has become a global trend (1). Long-term hospitalization has been identified as being problematic and is increasingly regarded as a practice that should be avoided. Mattes et al. have suggested that long-term hospitalization might not reduce subsequent admissions and does not clearly improve a patient's adaptation to society or reduce his/her psychopathology (2). Researchers have also pointed out that the costs associated with treatment of patients inappropriately admitted to a hospital greatly surpass the costs of community-based care (3). Therefore, the prolongation of hospitalization should also be avoided from an economic perspective. Sood et al. found that long-term hospitalized patients had a significantly poorer functioning, especially in self-care and domestic skills, compared with discharged patients (4). This suggests that staying in a hospital's sheltered environment for long periods of time comes with the risk of deterioration of patients' living and coping skills. Moreover, one study found that short-term hospitalization does not lead to “revolving door”-type hospitalization, or to low-quality or fragmented care (5).

Psychiatric hospital stays are more commonly prolonged in Japan than in other countries, and the environment of hospitalization wards and the community surrounding the psychiatric hospital is unique. There are approximately four times more beds (269 beds vs. 68 beds per 100,000 people) for psychiatric patients in Japan compared to the Organisation for Economic Co-operation and Development (OECD) average. These hospital beds continue to be used for the recuperation of chronic-stage patients (1). This is one of the reasons for which the length of stay in psychiatric hospitals in Japan is excessively long compared to that in European and American countries. Much like other countries, Ministry of Health, Labor and Welfare in Japan recommends that hospital stays are shortened. In 2004, the government launched a policy for shifting from hospital treatment to community-based care. Since the launch of this plan, the number of hospitalization days of newly admitted psychiatric patients has shown a decreasing trend. However, there still are over 50,000 patients per year who remain hospitalized for more than a year. In 2014, the government launched a policy that aimed to discharge patients from psychiatric wards within 1 year (6). It is becoming an increasingly important challenge to identify factors associated with longer hospitalization in Japan, where hospital stays already tend to be prolonged, and to consider actions to counter this problem.

It is important to focus on the factors that cause prolonged hospitalization and to continue to implement programs and actions designed to shorten hospital stays. Although it seems to be difficult to define which factors result in longer hospital stays, as Newman et al. have suggested that length of stay is likely to be multifactorially determined (7), attempts have been made to identify the factors related to the length of stay in psychiatric hospitals. For example, previous work has found that an older age is associated with a longer hospital stay (8–12). It is still unclear how sex is related to the length of hospital stay (7, 8, 13, 14). Psychotic disorder and mood disorder have been associated with longer stays (7, 12, 15–17), while personality disorder (11, 18), substance abuse (11, 13, 18), and adjustment disorder (18) have been associated with shorter stays.

Broderick et al. have reported that the number of episodes of physical violence during hospitalization lengthens hospital stay (19). On the other hand, Greenfield et al. have reported that violence itself is not a predictive factor for hospitalization (16). There are clear differences between reports on how violence is related to the length of hospital stay, and no uniform views have yet been achieved. From our clinical experience, violent patients seem to cause panic among healthcare staff, prevent therapeutic interventions, have more prominent mental symptoms, and narrow their post-discharge options. Therefore, we included the history of violent behavior in the factors potentially related to length of stay.

We hypothesized that violent behavior prior to admission is an important factor that affects the occurrence of discharge, and that patients with violent behavior continue to stay in the hospital for longer periods than patients without violent behavior. The purpose of this study was to explore factors that affect discharge and to evaluate the likelihood of hospital discharge to a community care setting based on a suite of demographic and clinical factors, with an emphasis on violent behavior.

## Methods

### Study Design and Setting

This retrospective cohort study was carried out at Tokyo Metropolitan Matsuzawa Hospital, Setagaya Ward, Tokyo, Japan. Tokyo Metropolitan Matsuzawa Hospital is a large-scale and long-established mental hospital with 808 psychiatric beds. The inpatient facility comprises 22 wards equipped with diverse functions. According to the 2018 statistics, the hospital had ~93,600 outpatients and returning patients per year, with 2,700 inpatients (20, 21). Patients with relatively severe symptoms are more likely to visit its outpatient clinic for treatment than clinics and private psychiatric hospitals. Patients may be admitted voluntarily at their own request. However, there also are cases where obtaining consent for admission is difficult, due to the influence of diverse psychiatric symptoms. In Japan, these patients may be admitted, even without their consent, after undergoing examination by mental health doctors designated by the Minister of Health, Labor and Welfare, if they are judged as requiring hospitalization, and if family members give their consent. This involuntary admission is specified under the Act on Mental Health and Welfare for the Mentally Disabled (22).

Upon involuntary admission, the doctor in charge specifies the planned treatment and the expected duration of hospitalization according to a predetermined format, explains it to the patient or family, obtains their consent, and stores it in their medical record. The planned lengths of hospitalization usually do not exceed 1 year. Patients who deviate from the plan and exceed the planned hospitalization duration undergo a review by a multidisciplinary team comprising physicians, nurses, and social workers.

### Participants

We selected subjects from the 4,729 patients who had been admitted to the Department of Psychiatry in Tokyo Metropolitan Matsuzawa Hospital from December 1, 2015 to November 30, 2017. We enrolled participants who, despite being hospitalized for medical care and protection, had exceeded their initially anticipated hospitalization period and had undergone a review by an inter-disciplinary team.

We selected only those patients who had been admitted involuntarily and had undergone an assessment of their needs toward discharge; namely, patients who deviated from the treatment plan established at admission and had exceeded the planned hospitalization duration. Patients discharged within the anticipated period were not selected as subjects. Furthermore, it was deemed inappropriate to include patients undergoing hospitalization for rest/recuperation purposes, wherein the patients could themselves choose the length of their hospital stay, or patients whose duration of hospitalization had already been predetermined.

**Figure 1:**
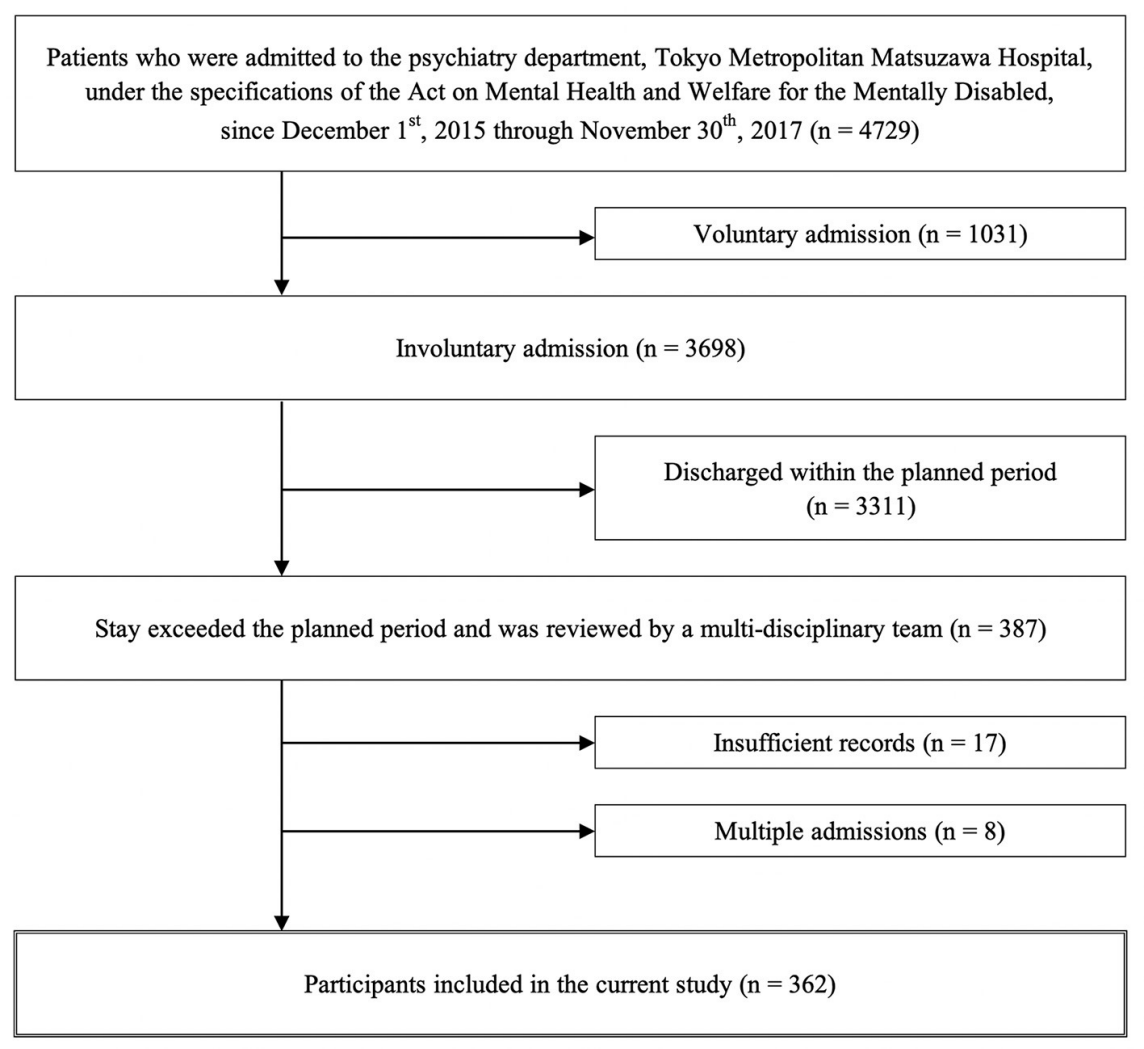
Patient flowchart describing the inclusion and exclusion criteria to identify target cases for the study.

The exclusion criteria were missing records, such as an insufficient summary (17 patients), as well as the second and subsequent admission of patients who had been hospitalized multiple times during the period (eight patients). Thus, 362 patients were selected as subjects for our study. The study inclusion and exclusion processes are shown in Figure 1.

### Procedures

We gathered data by retrospectively accessing electronic medical records. A structured data accessing, and coding protocol was used to ensure consistency in the order and method of data extraction. We employed each patient's summary at the time of discharge (if these contained insufficient information, we went back to summaries that had been written before those to confirm the information), and gathered demographic data, clinical data, data on the history of self-harm and other damaging behavior, and review by a multidisciplinary team.

### Outcomes and Measures

Discharge to community settings, such as home or other facilities, were defined as the outcome of this study. We excluded discharge due to transfer to other hospitals and death, because they could occur even under conditions where the adjustment of symptoms and the living environment had not been carried out sufficiently. Observations ended on January 14, 2020, and patients who stayed beyond this endpoint were categorized as censored subjects.

We investigated the effect of variables that have been reported to be associated with hospitalization in previous studies. Demographic data included age, sex, educational background (compulsory education, i.e., educated or not beyond Japan's legal requirement of middle school), lifetime marital history (presence/absence), and lifetime employment history (judged “Yes” if the subject worked full-time, or part-time, or even worked for a brief period) were collected.

Clinical data included the length of hospital stay, discharge destination, primary psychiatric diagnosis according to Tenth Revision of the International Statistical Classification of Diseases and Related Health Problems, the presence/absence of substance use disorder (treatment history for one or more of the following: alcohol, stimulants, marijuana, cocaine, organic solvents, designer drugs, and/or abuse of prescription drugs), intravenous sedation given/not given on admission, and implementation of electroconvulsive therapy (ECT) during hospitalization.

We reviewed the entire disease history in the summary. The reason for admission was noted in the disease history. If there was violent behavior coded on the Modified Overt Aggression Scale, we judged that “Violent behavior that led to the current admission” is present. Next, we categorized these as verbal violence, violence toward property and objects, self-harm, and physical violence. It is important to note that this includes not only physical but also verbal violence, violence toward property, and self-harm.

Data from the review by a multidisciplinary team were collected from the patients' electronic medical records. The review aimed to promote discharge by discussing the need for psychiatric hospitalization as well as measures and programs toward discharge and clarifying the planned period of hospital stay. Regarding the subjects' needs with respect to discharge, Tokyo Metropolitan Matsuzawa Hospital stipulates choosing multiple relevant items from the following: “Improvement of symptoms (presence of any unstable mental status that inhibits discharge),” “Insight of disease (patients' awareness of symptoms and treatment needs),” “Living skills (adequate daily living skills such as cooking, washing, and eating for life in community),” “Coping with symptoms (self-care ability to prevent relapse),” and “Preparation for living (housing, finances, food, and other life preparations).” The presence or absence of each of these items was collected as a binary variable. “Willingness to be discharged after the assessment” was a free description of the subject's hopes and wishes to be discharged. The presence or absence of requests to be discharged was also collected as a binary variable.

At the assessment, the multidisciplinary team reset the planned length of stay, but if the patient continued admission beyond that period, a review was held more than once during a single hospital stay. To prioritize early-stage assessments during hospitalization, we referred to the records of deliberations held for the first time.

### Statistical Analysis

First, we divided the subjects into two groups according to whether violent behavior had or had not led to the current admission (the Violent group and Non-violent group, respectively). We compared variables between groups using bivariate analysis. Chi-square tests were used to analyze between-group differences in sex, educational history, marital history, working history, substance abuse problems, intravenous sedation, ECT, and assessment of needs in prolonged-stay patients. Mann-Whitney U tests were used for between-group comparisons in age and length of stay.

Second, to investigate the influence of predictive factors for the length of hospitalization, we conducted survival analysis. The number of hospital days did not follow a normal distribution (mean = 301.5, standard deviation = 218.5, Shapiro-Wilk W = 0. 78, *P* < 0.001), even after a logarithmic transformation (mean = 2.4, standard deviation = 0.3, Shapiro-Wilk W = 0. 98, *P* < 0.001). All the explanation variables other than age were qualitative. We therefore established the occurrence of discharge to a community setting as the outcome, and used the Cox proportional hazard model to analyze the influence of multiple explanatory variables by taking the passage of time (length of stay) into consideration.

We performed Cox univariate analysis for each variable separately and investigated the relationships between the occurrence of discharge to the community and the latent predictive factors. Next, Cox multivariate analysis was used to assess the influence of independent variables on the discharge to community. We used age, sex, and variables that were significant at the *P* < 0.05 level in our univariate regression (diagnosis of schizophrenia, schizotypal and delusional disorders; implementation of ECT during admission; needs for improvement of symptoms; needs for insight of disease; needs for living skills; needs for coping skills with symptoms; and violent behavior that had led to admission), and included them as independent variables, using the forced entry method.

We used the correlation matrix table for all the variables to confirm that there were no combinations of variables that showed high correlation coefficients (above 0.9) to assess multi-collinearity of the variables to be entered into the multivariate analysis. We also observed a log-log graph of qualitative variables that were finally entered into the model and confirmed that proportional hazard assumptions had been maintained. IBM SPSS Statistics for Mac, version 23.0 (SPSS Inc., Chicago, IL, USA) was employed for all statistical analyses. The level of significance was set as α = 0.05.

### Ethical Considerations

This study was conducted in accordance with the recommendations of the Ethical Guidelines for Medical and Health Research Involving Human Subjects of the Japanese Ministry of Education, Culture, Sports, Science and Technology and the Ministry of Health, Labor, and Welfare. The need for informed consent was waived since this was a retrospective study. The research protocol was approved by the Tokyo Metropolitan Matsuzawa Hospital Ethics Committee (No. 26, FY2017) in accordance with the Declaration of Helsinki.

## Results

Table 1 summarizes the characteristics of the study subjects. Of the 362 subjects, 94 (26%) had violent behavior preceding hospitalization. The breakdown, based on multiple choice answers, was verbal violence for 28 subjects, violence toward property and objects for 28, self-harm for 22, and physical violence for 23. No significant differences were observed between the two groups in terms of demographic data, i.e., age, sex, educational background, career history, marital history, or history of substance abuse.

**Table 1:** Characteristics of the participants according to the presentation of violent behavior that led to the current admission.

**Variables**	**Total**	**Violent**	**Non-Violent**	P^*^
	**(*n* = 362)**	**(*n* = 94)**	**(*n* = 268)**	
**Demographic data**				
Mean age, yr (SD)	49 (16)	47 (17)	50 (16)	0.12
Male, *n* (%)	206 (57)	60 (64)	146 (55)	0.12
Less than high school diploma, *n* (%)	120 (33)	28 (30)	92 (34)	0.42
Lifetime marital history, *n* (%)	98 (27)	25 (27)	73 (27)	0.90
Lifetime working activity, *n* (%)	269 (74)	68 (72)	201 (75)	0.61
Substance abuse problem, *n* (%)	53 (15)	8 (9)	45 (17)	0.051
**Discharge destination**				
Discharged to community, *n* (%)	299 (83)	86 (91)	213 (80)	
Transferred to other hospital, *n* (%)	43 (12)	8 (9)	35 (13)	
Discharge due to death, *n* (%)	5 (1)	0 (0)	5 (1)	
Censored, *n* (%)	15 (4)	0 (0)	15 (4)	
**Length of stay**				
Median, days (25, 75%)	235 (158,356)	195 (157,290)	242 (159,378)	0.032
**Clinical characteristics**				
Primary diagnosis				
Schizophrenia, schizotypal, and delusional disorders, *n* (%)	266 (74)	67 (71)	199 (74)	
Mood disorders, *n* (%)	19 (5)	5 (5)	14 (5)	
Personality disorders, *n* (%)	12 (3)	2 (2)	10 (4)	
Intellectual disability, *n* (%)	29 (8)	12 (13)	17 (6)	
Other disorders, *n* (%)	36 (10)	8 (9)	28 (11)	
Intravenous sedation prior to admission, *n* (%)	205 (57)	60 (64)	145 (54)	0.10
Electroconvulsive therapy, *n* (%)	75 (21)	19 (20)	56 (21)	0.89
**Review by a multidisciplinary team**				
Needs for improvement of symptoms, *n* (%)	135 (37)	27 (29)	108 (40)	0.046
Needs for insight of disease, *n* (%)	139 (38)	28 (30)	111 (41)	0.046
Needs for living skills, *n* (%)	132 (37)	26 (28)	106 (40)	0.039
Needs for coping skills with symptoms, *n* (%)	170 (47)	39 (42)	131 (49)	0.22
Needs for preparation for living, *n* (%)	282 (78)	73 (78)	209 (78)	0.95
Willingness to be discharged after the assessment, *n* (%)	255 (70)	75 (80)	180 (67)	0.021

* *Chi-square tests were used for between-group comparisons in sex, educational history, marital history, working history, substance abuse problem, intravenous sedation, electroconvulsive therapy, and the assessment of needs in prolonged-stay patients; Mann-Whitney U-tests were used for between-group comparisons in age and length of stay*.

During the observation period, 299 subjects were discharged to the community, while 43 subjects remained in the hospital to which they had been transferred. There were also some subjects who died or continued their stay at Matsuzawa Hospital without being discharged and were censored.

The median length of stay in the Violent group was significantly shorter than the one observed in the Non-Violent group. The distribution of hospitalization days in the Violent and Non-violent groups was similar in the first quartile in both groups, but the median and third quartiles were larger in the Non-violent group. In other words, the distribution of hospitalization days in each group reflects a clear difference.

The most frequent diagnoses were those of schizophrenia and schizotypal and delusional disorders, accounting for 74% of the cases. Diagnoses of diseases other than schizophrenia were relatively rare.

There was no significant difference in occurrence between the two groups for subjects who received intravenous sedation on admission or ECT during hospitalization.

In terms of a review by a multidisciplinary team, “preparation for living” was selected as the item which most needed this.

The items assessed as being significantly more frequent in the Non-violent group were “Improvement of symptoms,” “Insight of the disease,” and “Living skills.” The “Willingness to be discharged after the assessment” was significantly more frequent in the Violent group.

Table 2 represents the results of the Cox univariate analysis, wherein a hazard ratio of greater than unity (one) suggests a higher likelihood of discharge, i.e., a shorter hospital stay. Diagnosis of schizophrenia and schizotypal and delusional disorder; implementation of ECT during admission; the needs for improvement of symptoms; the needs for insight of disease; the needs for living skills; and the needs for coping skills with symptoms were all associated with a significantly lower likelihood of discharge, whereas violent behavior that led to the current admission was associated with a significantly higher likelihood of discharge. We decided to enter these variables into the final model.

**Table 2:** Cox proportional univariate hazard ratios.

**Variables**	Hazard ratio^*^ (95% CI)	***P***
Age	0.994 (0.987: 1.002)	0.14
Male	0.883 (0.702: 1.112)	0.29
Less than high school diploma	0.899 (0.706: 1.145)	0.39
Lifetime marital history	1.248 (0.960: 1.623)	0.098
Lifetime working activity	0.794 (0.612: 1.030)	0.082
Substance abuse problem	1.136 (0.822: 1.571)	0.44
Schizophrenia, schizotypal, and delusional disorders	0.764 (0.588: 0.992)	0.044
Mood disorders	1.601 (0.978: 2.622)	0.061
Personality disorders	1.499 (0.796: 2.823)	0.21
Intellectual disability	1.282 (0.850: 1.933)	0.24
Intravenous sedation prior to admission	0.974 (0.774: 1.225)	0.82
Electroconvulsive therapy	0.725 (0.543: 0.969)	0.030
Needs for improvement of symptoms	0.710 (0.559: 0.901)	<0.010
Needs for insight of disease	0.693 (0.546: 0.880)	<0.010
Needs for living skills	0.658 (0.517: 0.839)	<0.010
Needs for coping skills with symptoms	0.769 (0.612: 0.967)	0.025
Needs for preparation for living	0.844 (0.644: 1.108)	0.22
Willingness to be discharged after assessment	1.267 (0.978: 1.642)	0.073
Violent behavior that led to the current admission	1.535 (1.189: 1.981)	<0.010

* *Hazard ratios of > 1 indicate increases in the probability of discharge*.

Table 3 represents the results of the proportional hazard analysis via the forced entry method. The results of the model Chi-square test revealed a significance level of *P* < 0.01. Violent behavior that led to current admission was associated with a significantly higher likelihood of discharge. The variable “Needs for living skills” was close to reaching a significant association with a lower likelihood of discharge.

**Table 3:** Cox proportional multivariate hazard ratios.

**Variables**	Hazard ratio^*^ (95% CI)	***P***
Age	0.995 (0.987: 1.002)	0.16
Male	0.987 (0.767: 1.251)	0.92
Schizophrenia, schizotypal, and delusional disorders	0.828 (0.628: 1.092)	0.18
Electroconvulsive therapy	0.809 (0.592: 1.105)	0.18
Needs for improvement of symptoms	0.808 (0.622: 1.050)	0.11
Needs for insight of disease	0.873 (0.637: 1.197)	0.40
Needs for living skills	0.755 (0.570: 0.999)	0.050
Needs for coping skills with symptoms	1.076 (0.803: 1.442)	0.62
Violent behavior that led to the current admission	1.428 (1.101: 1.854)	<0.010

* *Hazard ratios of > 1 indicate increases in the probability of discharge*.

## Discussion

In contrast to our hypothesis, in the subjects of our study, violent behavior prior to admission was associated with a significantly higher likelihood of discharge. In other words, it did not contribute significantly to prolonged hospitalization in patients who deviated from the treatment plan and had exceeded the planned hospitalization duration. The univariate analysis revealed that “Improvement of symptoms,” “Insight of the disease,” and “Living skills” were found to be significantly more frequent in the Non-violent group. The violent group more frequently expressed a wish to be discharged. The distribution of hospitalization days in the Violent and Non-violent groups was similar in the first quartile of both groups, but the distribution was larger in the median and third quartiles of the Non-violent group. The Cox proportional hazard model demonstrated an association between the violent behavior that led to the current admission and a significantly higher likelihood of discharge. The association between the “Needs for living skills” and the duration of hospital stay was close to reaching significance; other factors identified by previous studies (such as age, sex, educational background, career history, marital history, diagnosis) were not significantly associated with stay duration.

Participants in the current study were long-stay patients who had exceeded the planned hospitalization duration. The length of stay in European and American countries is relatively short, and many previous studies that have investigated the association between violence and length of stay have mainly focused on acute care settings. Greenfield et al. observed a mean duration of 14.9 days in university-based, short-term inpatient psychiatric units (16). Di Lorenzo et al. found a mean duration of 10.38 days in a public psychiatric ward (23). Comparing patients in the psychiatric inpatient units of a large urban county hospital with over 60 days of hospitalization to those with <30, Cheng et al. observed that factors associated with extended length of stay included older age, cognitive impairment, higher number of medical conditions requiring medication, and violence during hospital stay (9). However, a relative paucity of knowledge about patients with hospitalizations of longer than several months remains. Our results may help to address the gap in the literature by documenting how violence relates to community transitions in the population with long hospital stays. Moreover, we only targeted involuntarily admitted patients who stayed longer than planned, and underwent review by a multidisciplinary team to exclude the patients who were discharged either based on their own will or in compliance with a pre-established protocol, regardless of their conditions. In this way, we could isolate the influence of various factors characteristic of the patients themselves on their lengths of hospital stays.

The results of the univariate analysis between the Violent and Non-Violent groups may partially explain the characteristics of patients who deviated from the treatment plan and had exceeded the planned hospitalization duration for whom hospital treatment had been initiated after incidents of violence; despite violent tendencies, these patients tend to have relatively mild symptoms, appropriate life skills, and may be able to show insight and express a wish to be discharged. They may be able to be discharged relatively early as long as their violent tendencies and impulsiveness improve during acute treatment. However, this result does not completely rule out the observer bias that that violence may result in lower estimates of needed support. This finding overlaps with those of Wolff et al., who noted that risks posed to other people tend to result in shorter hospitalization, and stabilization of acute crises during the first days of stay might prompt early discharge (13). Most previous studies have examined violence during hospitalization, but this study only examined violence prior to hospitalization. As violence is recognized relatively easily and is liable to be mentioned as a problem by the people surrounding the individual in question, it can trigger admission. Our findings recommend caution in using violence and impulsiveness observed during the acute stage to predict the difficulty of long-term treatment.

The distribution of hospitalization days in the Violent/Non-violent groups indicate a clear difference. It may explain that the impact of other factors other than violence progressively exceed that of violence as the length of hospital stay increases. In a study that divided hospital stay into durations of less or more than 36 days, Di Lorenzo et al. hypothesized that aggressiveness itself could justify the difficulty in discharging patients, especially when it contributes to a vicious cycle of aggressive escalation (23). However, our study provides new findings that the effects of aggressiveness may not last past several months. Although our study could not verify this implication, further study of relatively short stay patients may be warranted to explore how the heterogeneous effects of violence vary with the length of hospital stay.

The association between “Needs for living skills” and the length of hospital stay was close to reaching significance in the multivariate analysis. The factor of living skill may become more significant as the length of hospitalization increases. This finding is particularly important for promoting discharge support to community settings in Japan, where prolonged hospital stays beyond a few months are problematic. In our study, violent tendency was not associated with further prolonged length of stay. It may explain that the prolonged hospitalization in Japan does not necessarily imply intention to isolate the patients from a security point of view. The OECD reported that Japanese hospital beds contain many beds for long-term inpatients that are not classified as psychiatric wards in European and American countries (1); our results may be related to this peculiarity of Japan. Okayama et al. also point out that beds which should be classified as community beds are still classified as “long-stay beds” of hospitals, due to the lack of public funding for private hospitals (24). There may be a rather large problem associated with institutional issues that are hindering patient transition back into the community. As Zhang et al. point out (25), this may indicate the need for the government to take initiatives to remove economic and social obstacles to the development of community support services.

The factors identified by previous studies, such as age, sex, educational background, career history, marital history, and diagnosis, were not presently found to be significantly associated with stay duration. However, it is necessary to consider that our survey did not target patients for whom treatment had proceeded smoothly; we only targeted long-stay patients whose symptoms complicated the management of treatment, whose discharge dates were consequently adjusted, and were required to undergo review by a multidisciplinary team. The demographic profile of each patient's background information, such as age, sex, and diagnosis, may not associate with the length of hospital stays in a population longer than several months.

### Limitations

The present study was subject to several limitations. First, although we have referred to the results of review by a multidisciplinary team by certified professionals, such as physicians, nurses, and social workers, we did not use a standardized assessment scale. This may have created an inter-reviewer bias. A standard assessment scale should therefore be used in future studies. For example, following previous studies, we would be able to use the Positive and Negative Syndrome Scale (PANSS) to assess symptoms, Social Behavior Scale (SBS) to assess social behavior, and Global Assessment of Functioning (GAF) for comprehensive assessment. Second, our survey was performed at a single institution. To verify whether our findings could be applied to other general clinical situations, a survey that expands the forum of research to other institutions is warranted. In addition, the length of hospitalization in Japan is much longer than that in European and American countries, and the legal system is also different. It should be noted that this is an exploratory study and may not be immediately generalizable to psychiatric wards in European and American countries. Third, this study did not target patients who had been discharged according to the initial plan, compromising the generalizability of our findings to participants who were discharged after a relatively short period. We may assess how each factor affects hospitalization in greater detail by expanding the range of selected subjects to patients who underwent treatment as planned and received no reviews by a multidisciplinary team and comparing those results to those presently reported. In addition, it may enable us to confirm in greater detail that the heterogeneous effects of violence vary with the length of hospital stay, as suggested in this study.

## Conclusion

Violent behavior prior to admission did not influence prolonged hospitalization among patients who deviated from the treatment plan and had exceeded the planned hospitalization duration. We recommend caution in using violence and impulsiveness observed during the acute stage to predict the difficulty of long-term treatment. The impact of factors such as living skills may become progressively greater than that of violence as the length of hospital stay increases. Further research that considers relatively patients of relatively shorter hospital stays may be needed to explore the heterogeneous effects of violence, as such effects may vary according to the length of patient's stay.

## Data Availability Statement

The datasets generated and/or analyzed during the current study are available from the corresponding author on reasonable request.

## Ethics Statement

The studies involving human participants were reviewed and approved by The Tokyo Metropolitan Matsuzawa Hospital Ethics Committee (No. 26, FY2017). Written informed consent from the participants' legal guardian/next of kin was not required to participate in this study in accordance with the national legislation and the institutional requirements.

## Author Contributions

HK designed the study, collected the data, undertook the statistical analysis, and interpreted the data. TO and NK designed the study and critically revised the first draft. HK wrote the first draft of the manuscript. All authors contributed to the article and approved the submitted version.

## Conflict of Interest

The authors declare that the research was conducted in the absence of any commercial or financial relationships that could be construed as a potential conflict of interest.

## Abbreviations

ECT: Electroconvulsive Therapy
OECD: Organisation for Economic Co-operation and Development.
